# Arsenic trioxide extends survival of Li–Fraumeni syndrome mimicking mouse

**DOI:** 10.1038/s41419-023-06281-2

**Published:** 2023-11-29

**Authors:** Jiabing Li, Shujun Xiao, Fangfang Shi, Huaxin Song, Jiaqi Wu, Derun Zheng, Xueqin Chen, Kai Tan, Min Lu

**Affiliations:** 1 Shanghai Institute of Hematology, State Key Laboratory of Medical Genomics, National Research Center for Translational Medicine (Shanghai), Ruijin Hospital affiliated to Shanghai Jiao Tong University School of Medicine, Shanghai, 200025 China; 2 School of Life Sciences and Biotechnology, Shanghai Jiao Tong University, Shanghai, China

**Keywords:** Cancer models, Tumour-suppressor proteins

## Abstract

Li-Fraumeni syndrome (LFS) is characterized by germline mutations occurring on one allele of genome guardian *TP53*. It is a severe cancer predisposition syndrome with a poor prognosis, partly due to the frequent development of subsequent primary tumors following DNA-damaging therapies. Here we explored, for the first time, the effectiveness of mutant p53 rescue compound in treating LFS-mimicking mice harboring a deleterious p53 mutation. Among the ten p53 hotspot mutations in IARC LFS cohorts, R282W is one of the mutations predicting the poorest survival prognosis and the earliest tumor onset. Among the six clinical-stage mutant p53 rescue compounds, arsenic trioxide (ATO) effectively restored transactivation activity to p53-R282W. We thus constructed a heterozygous *Trp53* R279W (corresponding to human R282W) mouse model for the ATO treatment study. The *p53*^R279W/+^ (W/+) mice exhibited tumor onset and overall survival well mimicking the ones of human LFS. Further, 35 mg/L ATO addition in drink water significantly extended the median survival of W/+ mice (from 460 to 596 days, hazard ratio = 0.4003, *P* = 0.0008). In the isolated tumors from ATO-treated W/+ mice, the representative p53 targets including *Cdkn1a*, *Mdm2*, and *Tigar* were significantly upregulated, accompanying with a decreased level of the proliferation marker Ki67 and increased level of apoptosis marker TUNEL. Together, the non-genotoxic treatment of p53 rescue compound ATO holds promise as an alternative for LFS therapeutic.

## Introduction

Germline mutation on one allele of the *TP53* tumor suppressor gene can result in Li-Fraumeni syndrome (LFS), a hereditary condition characterized by the development of multiple cancer types, often at a young or middle age [1–3]. LFS individuals face a lifetime cancer risk of up to 80–90%, with approximately half of them developing cancer by the age of 30 years [4, 5]. Despite the significantly increased risk of cancer-related morbidity and mortality, clinical management for LFS families is predominantly the cancer screenings such as annual whole-body MRIs and the prevention measures such as avoiding exposure to DNA-damaging agents and radiation [6–8]. Treatment options for LFS patients remain limited. The standard regimens involve DNA-damaging chemotherapies and radiotherapies, which often lead to subsequent primary tumors in LFS patients [1, 5]. The susceptibility to second primary tumors is expected since *TP53* functions as a haploinsufficient genome guardian. While mutant p53 rescue drugs, which restore tumor-suppressive function without causing DNA damage, are attractive alternatives, no such drugs have been approved for clinical use to date. Unfortunately, the development of mutant p53 rescue drugs for LFS treatment has received limited attention from the pharmaceutical industry due to the low prevalence of LFS (occurring in 1 in 5000 to 1 in 20,000 people worldwide) [9] and the formidable challenge in pharmacologically rescuing mutant p53, as evidenced by the lack of clinical trials for LFS treatment.

In laboratory settings, genetically engineered mouse models are valuable tools for studying LFS. In LFS individuals, one allele of *TP53* is inherently mutant, while the other allele remains wild-type. Heterozygous *Trp53* knockout mice partially resemble LFS patients in terms of tumor spectrum, tumor incidence, and survival [1, 10]. Soft-tissue sarcomas, osteosarcomas, and hematological malignancies are prevalent in both heterozygous *Trp53* mutant mice and LFS patients [6, 11–13]. Additionally, the high incidence of breast cancers in LFS patients is commonly observed in female p53 heterozygous mice of the BALB/c strain [14]. Moreover, approximately 50% of heterozygous *Trp53* knockout mice die from spontaneous tumors before 18 months of age [15], which parallels the median survival of approximately 50 years in LFS individuals [16]. However, due to the long lifespan of heterozygous *Trp53* mutant mice, a study on the efficacy of pharmacological treatment for LFS-mimicking mice is a long-term project.

Rescuing mutant p53 in LFS-mimicking mouse models is challenging due to the complicated p53 inactivation mechanisms and diverse functional consequences caused by the various p53 mutations [17]. For instance, the six classic p53 hotspot mutations, found in both somatic and germline cells, are located in the DNA binding domain (DBD) and can be classified as DNA-contacting mutations that affect DNA-binding residues, or structural mutations that affect structure-maintaining residues and cause protein unfolding [17, 18]. Based on the diversities of the p53 inactivation mechanism and functional consequences, a one-size-fits-all compound that can restore wild-type function to all p53 mutants should not exist [18]. Therefore, p53-rescue treatments in LFS need to differentiate mutations and, ideally, experimentally test the rescue effectiveness on the interested mutation before patient treatment.

Developing rescue compounds for mutant p53 is a highly challenging task due to the lack of actionable pockets in p53 and the need for a function-restoration mechanism of action (MoA) [17]. To date, there have been reports of over twenty generic mutant p53 rescue compounds, with six entering clinical trials, including ATO, APR-246, PAT, COTI-2, PEITC, and Kevetrin [19]. While ATO and PAT are being used to rescue a set of rescuable structural p53 mutations based on their mechanisms and experimental validations [20–22], APR-246, COTI-2, PEITC, and Kevetrin are being tested for rescuing all of the p53 mutations in laboratory and clinical settings. Therefore, for these six rescue compounds that have straightforward clinical trial potential in LFS patients, it is crucial to experimentally confirm their effectiveness in rescuing the specific p53 mutation detected in an LFS patient before clinical treatment.

In this study, we aimed to evaluate the efficacy of pharmacological rescue of mutant p53 using an LFS-mimicking mouse model. We initially differentiated the ten LFS p53 hotspot mutations based on their impact on prognosis using a large LFS cohort. Subsequently, we assessed the effectiveness of the six clinic-stage mutant p53 rescue compounds in rescuing the highly deleterious LFS hotspot mutations R282W and Y220C. With these validations, we constructed a heterozygous *Trp53* R279W (corresponding to human hotspot mutation R282W) knock-in mouse model and found that ATO significantly extended the overall survival of these LFS-mimicking mice, accompanied by reactivation of the p53-R279W. Our study provides a non-genotoxic pharmacological treatment approach for LFS, a disease with high unmet clinical needs.

## Results

### Prognosis of LFS individuals with diverse p53 mutations

We compiled the information on LFS cases from the International Agency for Research on Cancer (IARC) database (http://www-p53.iarc.fr/), which comprised 4455 records. Among these, 2836 cases were confirmed as germline carriers of *TP53* mutations (Supplementary Fig. S1A). The mutation and tumor spectrums were analyzed for these 2836 confirmed cases. All of the top ten p53 mutations, herein termed as hotpot mutations, in the 2836 cases are missense mutations, with nine of them located within the DNA binding domain (Supplementary Fig. S1B, 102–292 amino acids). R337H is a unique hotspot mutation, hitting the tetramerization domain of p53. Common tumors found in the LFS cohort include breast cancer, soft tissue sarcoma, brain, etc (Supplementary Fig. S1C).

**Fig. 1:**
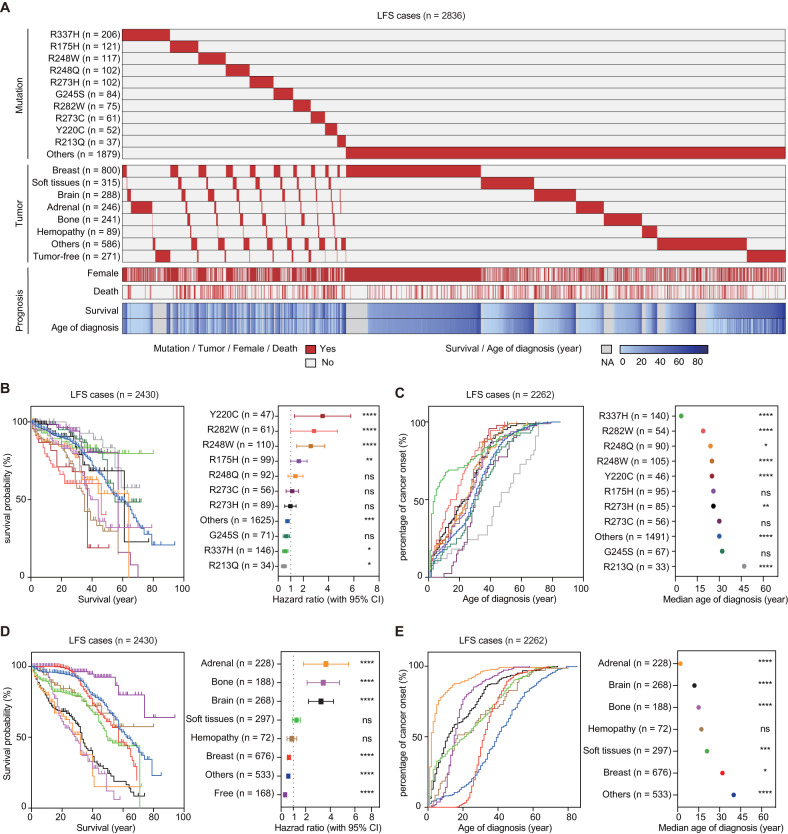
Prognosis of LFS individuals with diverse p53 mutations. **A** Landscape of p53 mutations, tumor types, and clinical prognosis in LFS cases. Data compiled from the IARC p53 germline mutation dataset is shown, including confirmed LFS individuals (*n* = 2836). **B** Kaplan–Meier survival curves and log-rank statistics of LFS cases harboring the 10 p53 hotspot mutations. Hazard ratios (HR) are calculated by comparing the survival of cases harboring the indicated mutation with the ones without this mutation. Confirmed germline carriers with the age of diagnosis and the age of follow-up were included (*n* = 2430). **C** Curve of cancer-onset age and the analysis of the median age of cancer onset for the LFS cases with indicated p53 mutations (*n* = 2262). **D** Kaplan–Meier survival curves and log-rank statistics of the indicated LFS subcohorts with specific tumor types (*n* = 2430). **E** Curve of cancer-onset age and the analysis of the median age of cancer onset for the LFS cases with indicated tumor types (*n* = 2262).

We next investigated the association between p53 mutation and tumor types with prognosis (full profiles seen in Fig. 1A). Among the 2430 cases with available p53 mutation and survival information, Y220C, R282W, R248W, and R175H were significantly associated with poorer survival compared to other mutations (Fig. 1B, *P* < 0.01, hazard ratio (HR) > 1). Among the 2262 cases with additional information on the age of cancer diagnosis, R337H, R282W, R248Q, R248W, and Y220C were the top mutations associated with the earliest tumor onset (Fig. 1C, *P* < 0.0001). Regarding the correlation between tumor types and prognosis, LFS cases with adrenal gland, bone, and brain showed significantly worse survival and earliest tumor onset, as compared to other subcohorts (All *P* < 0.0001, Fig. 1D, E). Overall, the median survival and median age of tumor onset in the LFS cohort were 57 and 29, respectively (Supplementar Figs. S1D, E).

In conclusion, LFS individuals with different p53 mutations exhibit highly diverse prognosis, with R282W, Y220C, and R248W being identified as the most deleterious LFS mutations.

### Screening of rescue compounds for the LFS p53 mutations

**Fig. 2:**
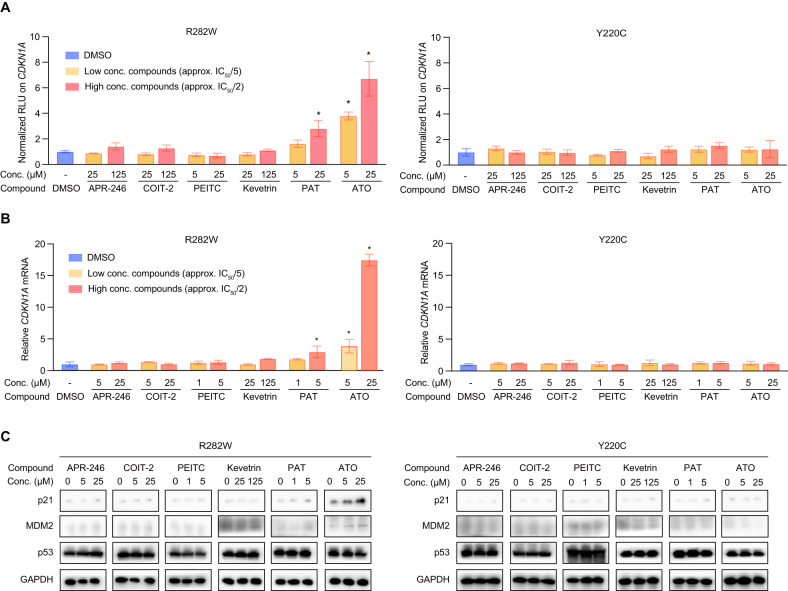
Screening of rescue compounds for the LFS p53 mutations. **A** Luciferase reporter assay for transactivation activity of LFS p53 mutation R282W and Y220C on the *CDKN1A* promoters. H1299 cells were treated with six indicated clinical-stage p53-rescue compounds at two concentrations (approx. IC_50_/5 and IC_50_/2 in H1299 cells) for 24 h. Bar graphs show the normalized RLU (relative light units). **B** Quantitative PCR (qPCR) determination of relative mRNA levels of *CDKN1A* upon treatment with indicated compounds and concentrations in U937 cells transfected with p53-R282W or p53-Y220C for 24 h. **C** Immunblotting for protein levels of *CDKN1A and MDM2* upon treatment with the indicated compounds and concentrations in U937 cells for 48 h. Bars represent mean ± SD, unpaired two-tailed Student’s *t* test, *n* = 3, *P < 0.05.

We then proceeded to investigate the rescue effectiveness of the six clinical-stage mutant p53 rescue compounds (APR-246, COTI-2, PEITC, Kevetrin, PAT, and ATO) on the deleterious LFS p53 mutations R282W and Y220C. Most if not all of the six compounds have been reported to action through increasing thermostability of structural p53 mutants. R248W was thus excluded in the investigation since it is a well-established DNA-contacting mutation, rather than a structural mutation [17, 23]. In the luciferase reporter assay conducted in H1299 cells [24], ATO significantly enhanced the transactivation activity of R282W on the *CDKN1A* promoter by approximately 3.8 times and 6.7 times at the two optimized concentrations (approx. IC_50_/5 and IC_50_/2) (Fig. 2A, left panel, both *P* < 0.05, *n* = 3). PAT also demonstrated rescue activity, increasing the transactivation activity of R282W on the *CDKN1A* promoter, although less effectively than ATO. The same results were observed when assaying the transactivation activity of R282W on the *MDM2* promoter (Supplementary Fig. S2A, left panel, *n* = 3). In the same treatment conditions, none of the remained four compounds significantly restored transactivation activity to R282W (Fig. 2A and Supplementary Fig. S2A, left panels; approx. IC_50_/5 and IC_50_/2 concentrations were used for each compound, *n* = 3). Interestingly, all six clinical-stage rescue compounds, including ATO and PAT, failed to significantly restore transactivation activity to the other deleterious LFS hotspot mutant p53-Y220C in this assay (Fig. 2A and Supplementary Fig. S2A, right panels, *n* = 3).

In the quantitative PCR (qPCR) experiment, only PAT and ATO showed effectiveness in significantly upregulating mRNA of the representative p53 targets, with increases ranging from 3.9 to 17.4 times for *CDKN1A*, and 3.5 to 9.3 times for *MDM2*, in U937 cells transfected with p53-R282W (Fig. 2B and Supplementary Fig. S2B, left panels, *n* = 3). In isogenic U937 cells transfected with p53-Y220C, none of the six tested rescue compounds significantly upregulated the mRNA levels of *CDKN1A* or *MDM2* (Fig. 2B and Supplementary Fig. S2B, right panels, *n* = 3). Thus, these qPCR results align with the observations from the luciferase assay.

We next examined the protein levels of p53 targets upon compound treatments using immunoblotting. ATO treatment demonstrated a dose-dependent upregulation of the protein levels for representative p53 targets p21 and MDM2 in U937 cells transfected with p53-R282W (Fig. 2C, left panels). PAT exhibited slight effectiveness, and only at high dosage (Fig. 2C, left panels). Again, the other four compounds showed no rescue effectiveness in the current immunoblotting experiments. In addition, all six clinical-stage rescue compounds failed to significantly upregulate p21 and MDM2 in U937 cells transfected with p53-Y220C (Fig. 2C, right panels).

In summary, the clinical-stage ATO efficiently rescued the deleterious LFS mutant R282W, while showing no significant rescue activity for Y220C.

### Establishment of LFS-mimicking mouse model with p53-R279W

**Fig. 3:**
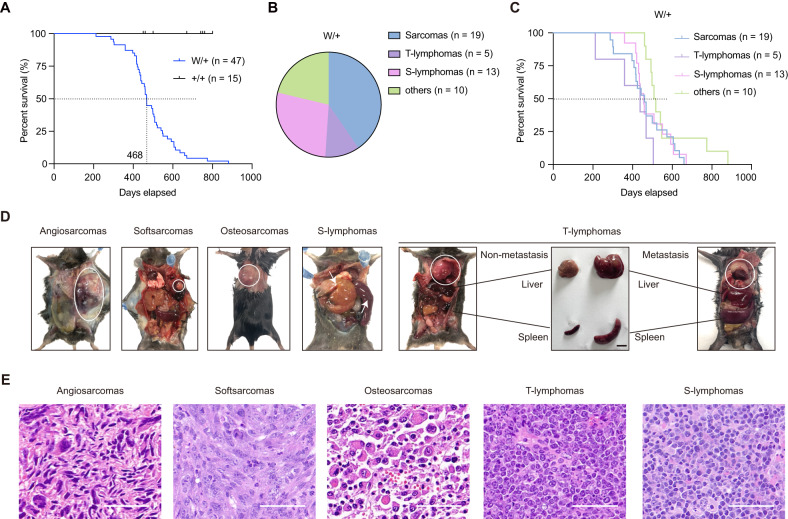
Establishment of LFS-mimicking mouse model with p53-R279W. **A** Kaplan–Meier survival curves of the mice harboring heterozygous p53 mutation R279W (p53^R279W/+^, W/+, *n* = 47) and the wild-type mice (p53^+/+^,+/+, *n* = 15). **B** Pie chart illustrating tumor spectrum of W/+ mice. Sarcomas (*n* = 19), T-lymphomas (*n* = 5), S-lymphomas (*n* = 13), and others (*n* = 10). **C** Kaplan–Meier survival curves of W/+ mice harboring different tumor types. **D** Macroscopic and phenotypic characteristics of the tumors spontaneously developed in W/+ mice. Scale bar, 1 cm. **E** H&E staining of the indicated tumors isolated from W/+ mice. Scale bar, 50 μm.

We thus focus on rescue compound ATO and the ATO-rescuable deleterious hotspot mutation R282W for further investigations. Initially, we utilized CRISPR/Cas9-based genome editing to generate p53 R279W mice (corresponding to amino acid 282 in humans) (Supplementary Fig. S3A). The heterozygous mutant mice (p53^R279W/+^, W/+, *n* = 47) that represent the LFS individual harboring the heterozygous p53 R282W mutant, exhibited a median survival of 468 days (Fig. 3A). This median survival largely mimics one of R282W LFS individuals (468 days in mice vs. approximately 46 years in humans, as shown in Fig. 1B). No survival difference was observed between the two genders of W/+ mice (Supplementary Fig. S3C, the number of males was 23 and females 24). The predominant spontaneous tumor types observed in W/+ mice were sarcomas (*n* = 19), thymic-lymphomas (T-lymphomas, *n* = 5), and spleen-lymphomas (S-lymphomas, *n* = 13) (Fig. 3B). The W/+ mice harboring different tumor types displayed similar overall survival (Fig. 3C).

We further specified the tumors spontaneously developed in the W/+ mice. The frequent occurrence of sarcomas in W/+ mice well mimics the high prevalence of sarcomas in LFS individuals harboring R282W (Fig. 1A). The sarcoma observed in W/+ mice included angiosarcoma, soft sarcomas, and osteosarcomas (Fig. 3D, left panel). Lymphomas also appeared frequently in W/+ mice, resembling the occurrence of hematological malignancies in LFS individuals (Fig. 1A). In W/+ mice, T-lymphomas occasionally metastasized, resulting in liver and spleen enlargement (Fig. 3D, right panel). Notably, we did not observe a high incidence frequency of breast cancer, which is the most common tumor type among LFS women, in our W/+C57BL/6N mice. This is consistent with the reported low rate of breast cancer in p53-defected C57 background mice and the high rate in p53-defected BALB/c background [14]. The representative gross morphology and hematoxylin and eosin (HE) staining images of the major spontaneously developing tumor types in W/+ mice are provided (Fig. 3D, E).

In conclusion, the constructed p53 W/+ mice largely mimic LFS individuals harboring the deleterious R282W mutation.

### ATO reactivates p53-R279W in LFS-mimicking mouse-derived cancer cell lines

Before initiating the long-duration in vivo study on W/+ LFS-mimicking mice, we validated the effectiveness of ATO in rescuing p53-R279W using cell lines derived from W/+ mice. We focused on sarcoma, the highly prevalent tumor type observed in both W/+ mice and LFS patients. Two cancer cell lines derived from sarcoma tissues freshly isolated from W/+ mice were used in our studies.

**Fig. 4:**
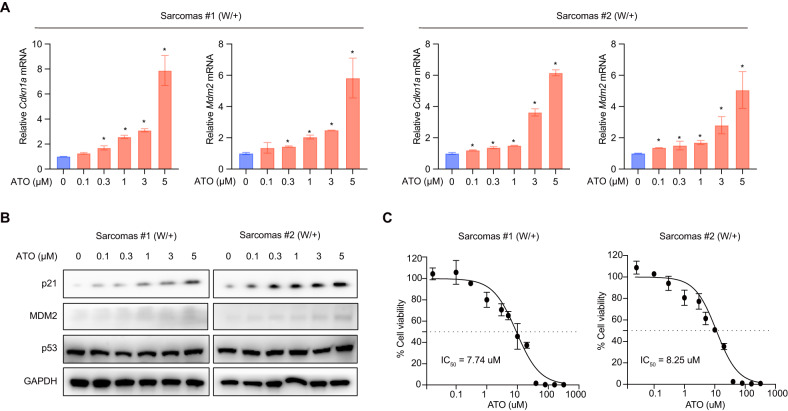
ATO reactivates p53-R279W in LFS-mimicking mouse-derived cancer cell lines. **A** qPCR determination of the mRNA levels of *Cdkn1a* and *Mdm2* in two sarcoma cell lines derived from W/+ mice. Cells were treated with ATO at indicated gradient concentrations for 24 h. **B** Immunoblotting of p21 and MDM2 protein in indicated cell lines treated with ATO at indicated gradient concentrations for 48 h. **C** Cell viability determination of indicated cell lines treated with indicated concentrations of ATO for 3 days. Bars represent mean ± SD, unpaired two-tailed Student’s *t* test, *n* = 3, *P < 0.05.

Upon ATO treatment, we observed a dose-dependent upregulation of mRNA levels of representative p53 targets including *Cdkn1a*, *Mdm2*, *Bax*, and *Puma* (Fig. 4A showing results for *Cdkn1a* and *Mdm2*; Supplementary Fig. S4A showing results for *Bax* and *Puma*, *n* = 3). Consistent with the mRNA evaluation, protein levels of Cdkn1a and Mdm2 were also detectably upregulated in the two W/+ sarcoma cell lines treated with ATO, in a dose-dependent manner (Fig. 4B). Furthermore, ATO effectively inhibited the proliferation of these two W/+ sarcoma cell lines, with IC_50_ of 7.74 µM and 8.25 µM, respectively (Fig. 4C, *n* = 3).

Based on these findings, ATO holds promise as a potential therapeutic option for the treatment of LFS-associated cancers.

### ATO extends survival of LFS-mimicking mouse with p53-R279W

**Fig. 5:**
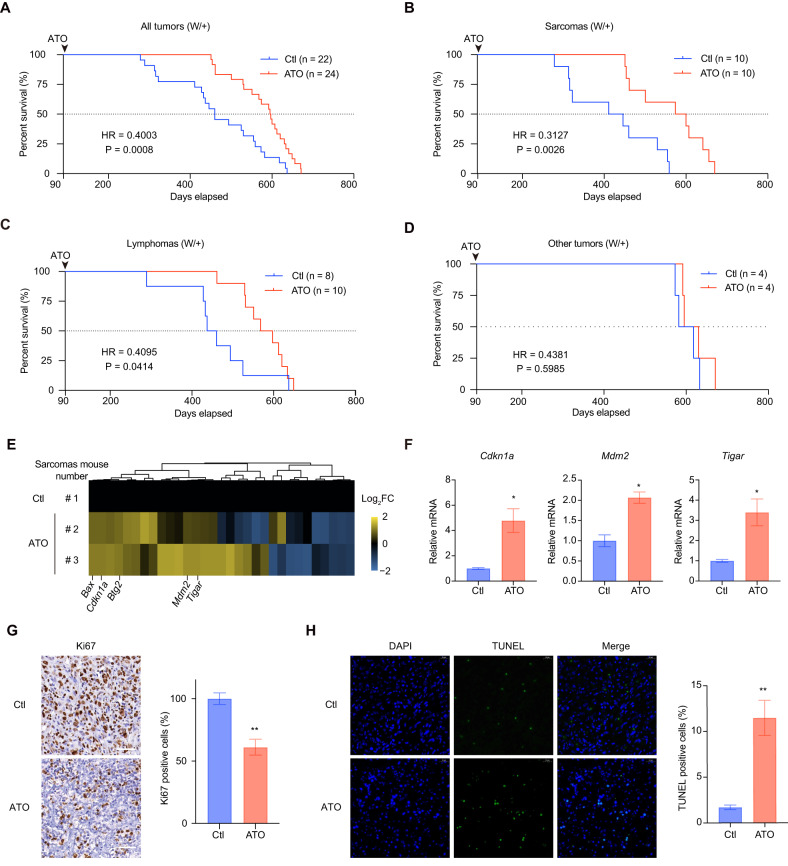
ATO extends survival of LFS-mimicking mouse with p53-R279W. **A**–**D** Kaplan–Meier survival curves of W/+ mice treated with or without ATO (35 mg ATO in 1 L drinking water) from day 90 until natural death were analyzed. **A** Mice with all tumor types (the mice number of ctl group was 22 and ATO group 24). **B** Mice with sarcomas (*n* = 10 per group). **C** Mice with lymphomas (the mice number of ctl group was 8 and ATO group 10). **D** Mice with other tumor types (*n* = 4 per group). **E** RNA-seq for sarcoma tissue isolated from ATO-treated or -untreated W/+ mice. Heatmap displays fold changes (FC) of mRNA levels of 32 confidently identified p53 targets compared to the untreated group. **F** qPCR analysis of relative mRNA levels of the indicated p53 targets *Cdkn1a*, *Tigar*, and *Mdm2*. **G** Representative immunohistochemical staining images of Ki67 and quantification of Ki67+ cells as percentages in the indicated group. Each dot in the plot represents the sum of the positive cells counted from six scopes randomly picked. Scale bars, 50 μm. **H** Representative immunofluorescence staining images of DAPI (blue) and TUNEL (green) and quantification of TUNEL+ cells as percentages in the indicated group. Each dot in the plot represents the sum of the positive cells counted from six scopes randomly picked. scale bars, 20 μm. Bars represent mean ± SD, unpaired two-tailed Student’s *t* test, *n* = 3, *P < 0.05. **P < 0.01.

Next, we conducted long-term treatments of W/+ mice with oral ATO administered on a daily basis (35 mg/L ATO in drinking water) starting from day 90 until natural death, followed by the designed experiments (Supplementary Fig. S5A). The treatment did not significantly alter the mice’s body weight (Supplementary Fig. S5B, *P* = 0.98, *n* = 3 per group). Encouragingly, ATO significantly extended the median overall survival of W/+ LFS-mimicking mice from 460 to 596 days (Fig. 5A, *P* = 0.0008, HR = 0.4003, the mice number of ctl group was 22 and ATO group 24). Regarding the survival of mice harboring sarcomas, ATO extended the median overall survival from 428 to 587 days (Fig. 5B, *P* = 0.0026, HR = 0.3127, *n* = 10 per group). Similarly, for the survival of mice harboring lymphoma, the median overall survival increased from 448 to 582 days (Fig. 5C, *P* = 0.0414, HR = 0.4095, the mice number of ctl group was 8 and ATO group 10). The other tumor types were observed in only a very small number of mice, making it impossible to determine the statistical effect of ATO on these tumor types (Fig. 5D, *n* = 4 per group).

To validate the reactivation of p53-R279W by ATO treatment in vivo, we performed RNA-seq analysis on sarcoma tissues obtained from ATO-treated and –untreated W/+ mice. Principal component analysis (PCA) was employed to assess the correlation among samples and the distribution was visualized in the principal component (PC) score plot. The plot revealed a relatively large difference between the ATO-treated and -untreated groups and a relatively smaller difference within the ATO-treated group (Supplementary Fig. S5C). The heatmap analysis of 32 confidently identified p53 targets from over 10 independent genome-wide datasets [25] demonstrated a successful rescue of mutant p53 by ATO, as evidenced by the global upregulation of these 32 targets, including well-studied ones such as *Cdkn1a*, *Tigar*, and *Mdm2* (Fig. 5E). In addition, qPCR analysis of these three p53 target genes confirmed their significant upregulation at the mRNA levels (Fig. 5F, *n* = 3).

Concurrently, we performed immunohistochemistry experiments using the proliferation marker, Ki-67, and immunofluorescence experiments using the apoptosis marker TUNEL. The positive rate of Ki67 cells was significantly lower, while the positive rate of TUNEL cells was significantly higher in the sarcomas tissues isolated from ATO-treated W/+ mice compared to the untreated mice (Fig. 5G, H, *P* < 0.05, *n* = 6). These results suggest that ATO effectively extends the survival of W/+ LFS-mimicking mice through mutant p53 reactivation.

## Discussion

LFS is a hereditary condition that occurs in approximately 1 in 5000 to 1 in 20,000 people worldwide [9]. This rarity partly explains why LFS treatment regimens have received limited attention from pharmaceutical companies, resulting in a scarcity of clinical trials for LFS. Among the 97,726 cancer-associated clinical trials registered on ClinicalTrials.gov by 2023, the NCT01981525 trial conducted in 2014, which utilized metformin, is the only intervention trial for LFS cancer patients. Furthermore, due to the long lifespan of the LFS-mimicking mouse model, the complicated inactivation mechanisms and diverse functional consequences resulting from various p53 mutations, and inconsistent reports on the effectiveness of mutant p53 rescue compounds (such as the extensively studied CP-31398 and APR-246 [26–30]), there are no reports on the efficacy of pharmacological rescue of mutant p53 in LFS-mimicking mouse model.

**Fig. 6:**
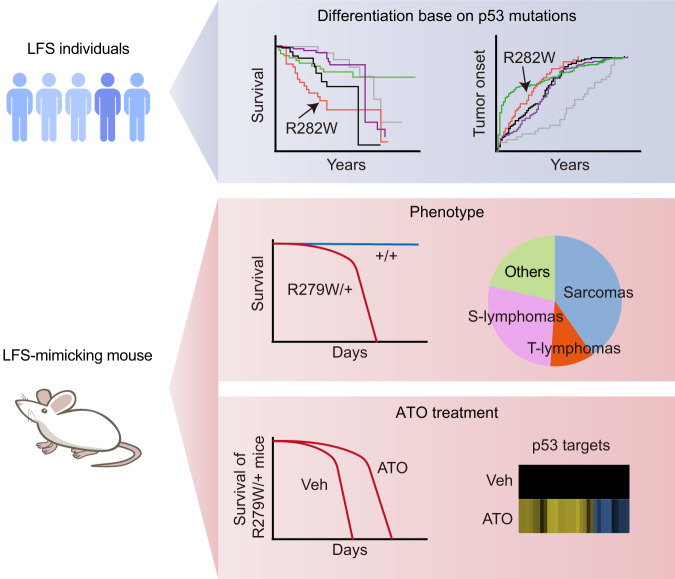
Summary of the Study. Top panel: LFS individuals harboring different p53 mutations exhibit diverse survival curves and tumor-onset curves. Middle panel: W/+ mice exhibit overall survival and tumor spectrum mimicking those of human LFS individuals. Bottom panel: ATO extended the survival of the W/+ LFS-mimicking mouse, accompanied by mutant p53 reactivation.

Here we aimed to address these gaps by proposing the use of non-genotoxic ATO as an alternative to the current DNA-damaging chemotherapy and radiotherapy, which are commonly employed for clinical LFS treatment but often lead to subsequent primary tumors. Initially, we analyzed p53 mutations in the IARC LFS cohort and found highly diverse survival and tumor onset among LFS cases harboring different p53 mutations (Fig. 6, top panel). Notably, R282W, Y220C, and R248W are among the LFS hotspot mutations associated with the poorest survival prognosis and the earliest tumor onset. Subsequently, we screened six clinical-stage mutant p53 rescue compounds and identified ATO as an effective rescue compound for the deleterious R282W, but not Y220C. Y220C represents a ‘warm spot’ mutation, positioned just beyond the spectrum of the six classical hotspot mutations in terms of mutation frequency. Our previous studies on the rescue of a comprehensive panel encompassing 25 frequent p53 mutations, alongside 800 common cancer-associated p53 mutations, as well as our present investigation, consistently illustrate the modest or negligible potency of ATO in rescuing Y220C [20, 31]. This outcome is unexpected, considering that p53-Y220C is a well-documented structural mutant, characterized by its unfolded and inactivated state at 37°C [32]. Remarkably, ATO exhibits remarkable efficacy in refolding structural p53 mutants, surpassing other established rescue compounds by orders of magnitude [20]. The precise mechanism underlying the suboptimal rescue potency of ATO for Y220C remains elusive, though its temperature-sensitive (TS) nature could offer a plausible explanation. Mutations located at the DNA-binding surface, such as R249S, are commonly non-TS variants due to their distorted DNA-binding interfaces in their folded states. As a result, ATO-mediated rescue efficiency is compromised for these mutants [20]. In contrast, structural mutations situated within the β-sandwich domain, like V272M, frequently display TS properties due to their intact DNA-binding surfaces in the folded conformation, rendering them susceptible to robust ATO-mediated rescue [31]. Y220C occupies a position within the β-sandwich domain, unlike DNA-binding surface mutations. This could imply a temperature-sensitive behavior, yet distinct from classic TS mutants, as Y220C’s positioning lies at the periphery of the β-sandwich rather than its core. Our unbiased assessment of 800 common cancer-associated p53 mutants previously established Y220C as a non-TS mutant [22, 31], emphasizing that even upon refolding through low-temperature treatment, p53-Y220C cannot be efficaciously restored in function. Consequently, this suggests that p53-Y220C does not effectively restore its function even when refolded by thermostabilizing rescue compounds such as ATO. Notably, a category of Y220C-specific rescue compounds has emerged, exploiting the unique Cys220 pocket within this mutation [32]. These compounds, distinct from generic rescuers like APR-246 and ATO, are designed to selectively target individual Y220C mutants [33–35]. Among these, PC14586 has entered clinical trials for patients harboring Y220C [34, 36]. Unfortunately, due to its limited accessibility and undisclosed molecular structure, our ability to evaluate its effectiveness in rescuing p53-Y220C remains restricted. In conclusion, our investigation highlights the inherent limitations associated with ATO [20], PAT [22], or low-temperature [22, 31] treatments in rescuing the non-TS structural mutation Y220C. Different from Y220C, R282W represents a structural mutation within the context of a temperature-sensitive (TS) phenotype [22, 31]. Here we performed in vitro studies on this mutant for its transactivation activity and target gene expression by using luciferase reporter assay and qPCR determination, revealing successful rescue of this mutant in two cancer cell lines expressing exogenous human mutant p53-R282W. Additionally, our investigations extended to two sarcoma cell lines derived from LFS-mimicking mice, which harbor endogenous mouse p53-R279W (equivalent to human p53-R282W). We further performed in vivo studies on this mutant for its target gene expression by using RNA-seq and qPCR determination, confirming that the endogenous mouse p53-R279W in spontaneous sarcoma tumors can be effectively rescued by ATO oral administration. Based on this rationale, we conducted experiments to determine the treatment efficacy of ATO in the W/+ mouse model by simply adding ATO to their drinking water. This mouse model partially mimics the survival and tumor spectrum observed in human R282W LFS (Fig. 6, middle panel). Encouragingly, oral ATO significantly extends W/+ mice survival (HR = 0.4003, *P* = 0.0008). The extended mouse survival was accompanied by the upregulation of p53 targets, and importantly, we did not observe significant side effects associated with ATO treatment (Fig. 6, bottom panel).

The notable extension of mouse survival through ATO treatment, achieved without inducing significant adverse effects in our study, holds particular significance. This is especially pertinent considering ATO’s established reputation as a toxic agent within clinical contexts, where its narrow therapeutic window (toxic concentration vs. effective concentration in the human body) has posed a substantial hurdle to the repositioning of ATO for clinical use. In the clinics, ATO is commonly administered in combination with all-trans retinoic acid (ATRA) for acute promyelocytic leukemia (APL) treatment, with a dosage of 0.15 mg/kg per day [37–39]. This dosage generated manageable side effects, primarily reversible grade I-II hepatic toxicity [37–39] and cardiovascular toxicity [38]. However, certain side effects, including QT interval (QTc) prolongation, pneumonitis, and neuropathic pain have emerged as dose-limiting concerns [38]. Increasing the dosage to 0.2 mg/kg per day caused a higher chance of dose-limiting side effects [38], highlighting the relatively constrained therapeutic range of ATO treatment. In the current investigation, we administered ATO at a concentration of 35 mg/L in the drinking water of mice. This dosage proved to be well-tolerated, as indicated by the absence of significant alterations in the body weights of ATO-treated mice. To the best of our knowledge, there exists no prior report of long-term ATO treatment in a spontaneous cancer mouse model for the purpose of survival assessment. Conversely, short-term ATO treatment has been widely documented for cancer inhibition in xenograft models, including our own studies [20, 31]. Remarkably, these xenograft investigations consistently employed an ATO dosage of 5 mg/kg, akin to the dosage utilized in our present study, accounting for a daily water intake of approximately 5 mL by a 25 g mouse.

Despite the observed survival benefits, it remains unclear whether these benefits are attributed to delayed cancer onset, slowed cancer progression, or both. Theoretically, these two mechanisms could be applied for cancer prevention in tumor-free LFS individuals and for cancer treatment in LFS cancer patients, respectively. Additionally, some p53 mutants have oncogenic gain-of-function (GOF), a phenotype reported to be associated with structural instability [40–42]. Since ATO has shown remarkable potency in enhancing the structural stability of p53 [20, 43], it would be worthwhile to investigate whether ATO can also abrogate oncogenic GOF of structural p53 mutants, in addition to restoring tumor-suppressive function, thereby contributing to the observed survival benefit in W/+ LFS mice.

Genetic predisposition toward mammary tumorigenesis has been previously documented [14, 44]. In our study involving p53-deficient heterozygous C57-background mice, we did not observe breast tumors among 47 tumor-bearing mice (Fig. 3B). It is noteworthy that the incidence of breast tumors in our model is considerably lower than the prevalence reported in Li-Fraumeni syndrome (LFS) families (Fig. 1A), where 800 cases of breast cancer were reported among a total of 2,836 cancer cases. Given the significantly elevated occurrence rate of breast cancer in LFS families, we acknowledge the limitations of our p53-deficient heterozygous C57-background model. To address this limitation and further explore the potential for LFS breast cancer treatment, we suggest employing BALB/c-background mice in subsequent studies. Notably, it has been reported that female p53-deficient heterozygous mice of this genetic background demonstrated a mammary carcinoma incidence of 55% [14].

Considering the lengthy testing periods and high costs associated with developing new anticancer drugs, repurposing the old drug ATO in a clinical trial for LFS patients could be a straightforward approach.

## Methods

### p53 germline mutation and clinical data

The germline mutation data came from the International Agency for Research on Cancer (IARC) TP53 Database (R20; accessed on 1 April 2021). Only cases that have been confirmed as germline carriers of TP53 mutation were included (*n* = 2836). In addition, the cases without the age of diagnosis and the age of the cases at the time of follow-up were removed for further analysis.

### Cell lines

HCT116 (ATCC, CCL-247), and H1299 (ATCC, CRL-5803) cells were purchased from ATCC. All cell lines were cultured at 37 °C in DMEM (Shanghai Basalmedia Technologies) supplemented with 10% FBS, 100 U/mL penicillin, and 100 mg/mL streptomycin. All cell lines were tested to be mycoplasma free.

### Chemicals

Eprenetapopt, APR-246 (Selleck Cat# S7724). COTI-2 (MCE Cat# HY-19896). Kevetrin hydrochloride (Topscience Cat# 66592-89-0). Potassium antimony tartrate, PAT (Sigma Cat# 244791). Arsenic trioxide, ATO (Sigma Cat# 202673). Phenethyl isothiocyanate, PEITC (Sigma Cat# 252492).

### Luciferase reporter assay

Luciferase reporter assay was performed as previously reported [24]. Briefly, H1299 cells were transfected with plasmids (p53-R282W-expressing plasmid or p53-R248W-expressing plasmid, luciferase reporter plasmid, Renilla plasmid) using FuGENE transfection reagent (Promega, E1960). After transfection for 24 h, the chemical was added, and cells were lysed the next day, followed by luciferase signal determination using a luciferase assay kit (Vazyme, DL101-01).

### Quantitative real-time PCR

RNA extraction was performed using the TRIzol reagent (Invitrogen) according to the manufacturer’s protocol. 1 μg RNA was reverse-transcribed using HiScript II Q RT SuperMix for qPCR (+ gDNA wiper) (Vazyme Biotech, R223-01) to generate cDNA. Real-time PCR was performed in duplicate using ChamQ SYBR qPCR Master Mix (Vazyme Biotech, Low ROX Premixed; Q331-02/03) and a ViiATM 7 Real-time PCR System (Applied Biosystems) under the following conditions: 10 min at 95 °C, followed by 40 cycles of 95 °C for 15 s and 60 °C for 60 s. The following qRT-PCR primers were used: CDKN1A_F GTCTTGTACCCTTGTGCCTC, CDKN1A_R GGTAGAAATCTGTCATGCTGG, PUMA_F ACGACCTCAACGCACAGTACG, PUMA_R TCCCATGATGAGATTGTACAGGAC, MDM2_F ATCAGGCAGGGGAGAGTGAT, MDM2_R CAATTCTCACGAAGGGCCCA, β-actin_F ACTTAGTTGCGTTACACCCTTTC, β-actin_R GACTGCTGTCACCTTCACCGT.

### Immunoblot

Cells were lysed with RIPA buffer (Biyuntian, P0013B) containing protease inhibitors. Lysates were cleared by 10,000 rpm centrifugation at 4 °C, and protein concentration was measured using the Bicinchoninic acid (BCA) method (Biyuntian, P0009). Equivalent lysates were separated by SDS-PAGE and electrotransferred onto nitrocellulose membranes (Bio-Rad) and blocked with TBS containing 0.1% Tween 20 (Sigma) and 5% skimmed milk for 30 min. The protein expressions were detected with the following antibodies: p21 (Cell signaling Cat# 2947), p21 (Santa Cruz Biotechnology Cat# sc-817), p53 (Abcam Cat# ab26), p53 (Abcam Cat# ab1101), β-actin (Santa Cruz Biotechnology Cat# sc-47778 HRP), Mdm2 (Sigma-Aldrich Cat# M4308), and Gapdh (ABclonal Cat# AC002).

### Generation of p53-R279W mutant mice

CRISPR/Cas9-mediated gene targeting was used to generate mouse models. For genotyping, mouse toes were lysed with mouse tissue lysis buffer (Vazyme, PD101-01) containing proteinase K for DNA extraction (incubation at 55 °C for 30 min and then inactivate proteinase K at 95 °C for 5 min). Primers used for PCR reactions to detect mutant alleles were GTT CCA CGA GTC CCG CC (forward primer) and GGC ATG CGA CTC TCC AGC CTT (reverse primer).

### Survival study of W/+ mice

For the survival study of W/+ mice, the mice were monitored twice weekly and euthanized when animals appeared moribund or when the largest palpable tumor reached 2 cm^3^ or ulcerated. On day 90 after birth, heterozygous (p53^R279W/+^, W/+) mice drink water daily with ATO (35 mg ATO in 1 L drinking water) until death.

### Isolation of primary tumor cells

Mice were euthanized with isoflurane and placed on the dissection pad. Separated the tumor from the organs and transferred to cold PBS kept on ice. Cut the tumor with scissors and remove the large tumor lump. Transfer into DMEM medium (supplemented with 10% FBS, L-glutamine, penicillin/streptomycin) and cells pass through a 40 mm nylon cell strainer (Falcon) to remove cell clumps. Spin down, resuspend the cells, and cultured in DMEM medium at 37 °C in a humidified 5% CO_2_ environment.

### Cell viability measurement

Primary tumor cells were seeded into a 96-well plate (5000 cells per well) for 24 h. Then, cells were treated with the indicated concentration of ATO. After 3 days of treatment, 10 μl of CCK-8 regent (CK04, DOJINDO) were added to each well and incubated for 1 h at 37 °C before being read on a Wallac EnVision plate reader (PerkinElmer). Readings of treated wells were normalized to untreated wells (set to 100% luminescence) and wells containing only media (set to 0% luminescence).

### Immunohistochemistry

Tumor tissues were fixed in 10% formaldehyde and paraffin-embedded. After dewaxing, rehydration, and antigen retrieval, sections were blocked with 3% BSA (G5001, Servicebio), followed by staining using rabbit polyclonal antibody Ki-67 (GB111141) and biotinylated goat anti-rabbit immunoglobulin (Servicebio Cat# GB23303) overnight at 4 °C, respectively. After staining using a secondary antibody (Dako, K5007) at 37 °C for 30 min, visualization was performed using a DAB detection kit (Dako, K5007) and Pannoramic 250FLASH (3DHISTECH). The quantification was done at 400× magnification (3 images per tumor) in five randomly selected areas using ImageJ software (NIH Image J system, Bethesda, MD).

### RNA-seq

Total RNA was extracted from tumor tissues using the Spin Column Animal Total RNA Purification Kit (Sangon Biotech, B518651), and mRNA was purified from 1 μg of the extracted RNA using the Illumina TruSeq RNA Sample Prep Kit (cat# FC-122-1001) to prepare sequencing libraries. The libraries were pooled and sequenced on the Illumina Novaseq 6000 platform, generating 150 bp paired-end reads. Raw reads were initially processed using FastQC (v0.10.1) for quality control, while adapter sequences and poor-quality reads were removed using Cutadapt (v1.9.1). Quality-filtered reads were aligned to the Mus musculus genome (GRCm38) using HISAT2 (v2.0.1). Gene-level read counts were generated from the aligned reads using HTSeq (v0.6.1). To perform the Principal Component Analysis (PCA), the prcomp function in R Studio was used. Finally, the heatmap was generated using R with pheatmap (v1.0.12).

### Statistics analysis

Statistical analyses were performed using Prism 9 (GraphPad), and an unpaired two-tailed Student’s *t* test with a 95% confidence interval under the untested assumption of normality was used to determine statistical significance. The standard deviation (SD) of the number of replicates was represented as error bars in the figures. The survival rate was calculated using the Kaplan–Meier analysis, and the significance of differences between survival curves was determined using the log-rank test. Hazard ratios (HR) and 95% confidence intervals (CI) were employed to assess the risk ratio of death. The significant differences between the two groups were noted by asterisks (*P < 0.05, **P < 0.01, ***P < 0.001, ****P < 0.0001).

### Supplementary information

Supplemental Figure Legends

supplemental figure 1

supplemental figure 2

supplemental figure 3

supplemental figure 4

supplemental figure 5

Original Data File

aj-checklist

Table S1, The IARC p53 germline mutation database, related to Figure 1.

Table S2 RNA-sequencing of mouse spontaneous tumors, related to Figure 5E

## Data Availability

This study’s raw and processed RNA-seq data are available at the Gene Expression Omnibus (GEO) under accession number GSE234883.
