# Supplementary material for: Arsenic trioxide extends survival of Li–Fraumeni syndrome mimicking mouse
Source: Cell Death Dis. 2023 Nov 29;14(11):783. doi: 10.1038/s41419-023-06281-2 (PMC10687230; doi:10.1038/s41419-023-06281-2)
Supplement: Supplementary file 1 — Supplemental Figure Legends [file 41419_2023_6281_MOESM1_ESM.docx]

**Figure S1. Prognosis of LFS individuals with diverse p53 mutations**

(A) Flowchart describing the analysis of the study. Confirmed cases, the individual has been tested for the presence of the p53 mutation and the mutation has been found.

(B) Positions of p53 germline hotspot mutations derived from the International Agency for Research on Cancer (IARC, R20). The x-axis represents the amino acid sequence of the p53 protein, while the y-axis displays the mutation rate.

(C) Tumor spectrum of IARC LFS cohort.

(D) Kaplan-Meier survival curve of confirmed LFS cases with survival information (n = 2,430).

(E) Curve of cancer-onset age of confirmed LFS cases with tumor (n = 2,262).

**Figure S2. Screening of rescue compounds for the LFS p53 mutations**

(A) Luciferase reporter assay for transactivation activity of LFS p53 mutation R282W and Y220C on the *MDM2* promoters. H1299 cells were treated with six indicated clinical-stage p53-rescue compounds at two concentrations (approx. IC_50_/5 and IC_50_/2 in H1299 cells) for 24 hours. Bar graphs show the normalized RLU (relative light units).

(B) qPCR determination of the mRNA levels of the *MDM2* upon treatment with indicated compounds and concentrations in U937 cells transfected with indicated p53 mutants.

Bars represent mean ± SD, unpaired two-tailed Student’s t-test, n = 3, *P < 0.05. **P < 0.01.

**Figure S3. Establishment of LFS-mimicking mouse model with p53-R279W**

(A) CRISPR/Cas9-based genome editing strategy used to construct the p53-R279W mutation.

(B) Pie chart depicting the gender distribution of W/+ mice. The number of females was 74 and males 79.

(C) Kaplan–Meier survival curves for male (n = 23) and female (n = 24) W/+ mice.

**Figure S4. ATO reactivates p53-R279W in LFS-mimicking mouse-derived cancer cell lines**

(A) qPCR determination of the mRNA levels of *Bax* and *Puma* in two sarcoma cell lines derived from W/+ mice. Cells were treated with ATO at the indicated gradient concentrations for 24 hours. Bars represent mean ± SD, unpaired two-tailed Student’s t-test, n = 3, *P < 0.05.

**Figure S5. ATO extends survival of LFS-mimicking mouse with p53-R279W**

(A) Schematic diagram illustrating the ATO treatment experiment *in vivo*.

(B) Body weight curves of ATO-treated and -untreated W/+ mice (n = 3 per group).

(C) Two-dimensional Principal Component Analysis (PCA) score plot for W/+ mice with sarcomas treated with or without ATO.

**TABLES**

Table S1 The IARC p53 germline mutation database, related to Figure 1.

Table S2 RNA-sequencing of mouse spontaneous tumors, related to Figure 5E.
