# Supplementary figures and images for: Arsenic trioxide extends survival of Li–Fraumeni syndrome mimicking mouse
Source: Cell Death Dis. 2023 Nov 29;14(11):783. doi: 10.1038/s41419-023-06281-2 (PMC10687230; doi:10.1038/s41419-023-06281-2)

# Supple 1

**A**

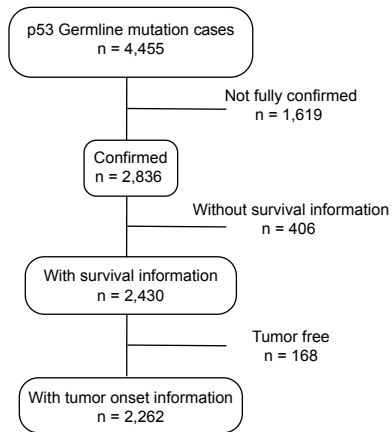

**C**

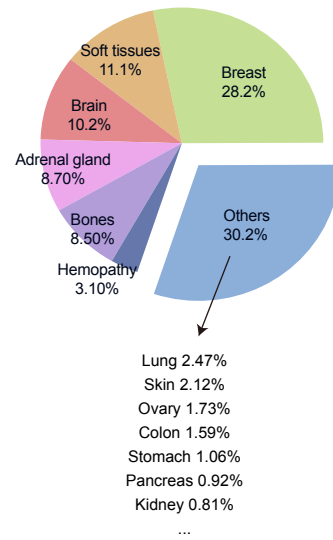

**D**

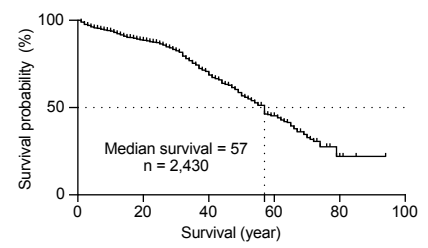

**E**

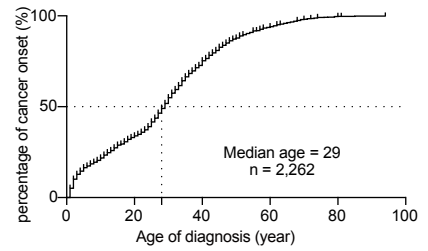

**B**

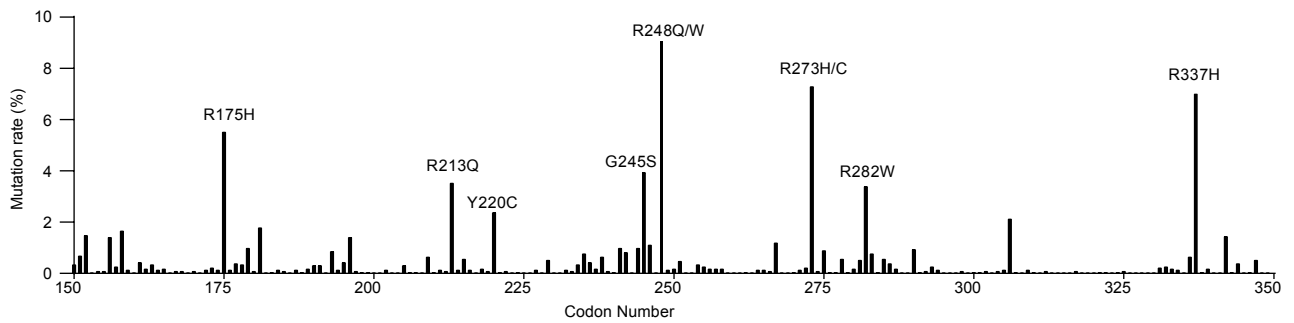

Supplement: Supplementary file 2 — supplemental figure 1 [file 41419_2023_6281_MOESM2_ESM.pdf]

# Supple 2

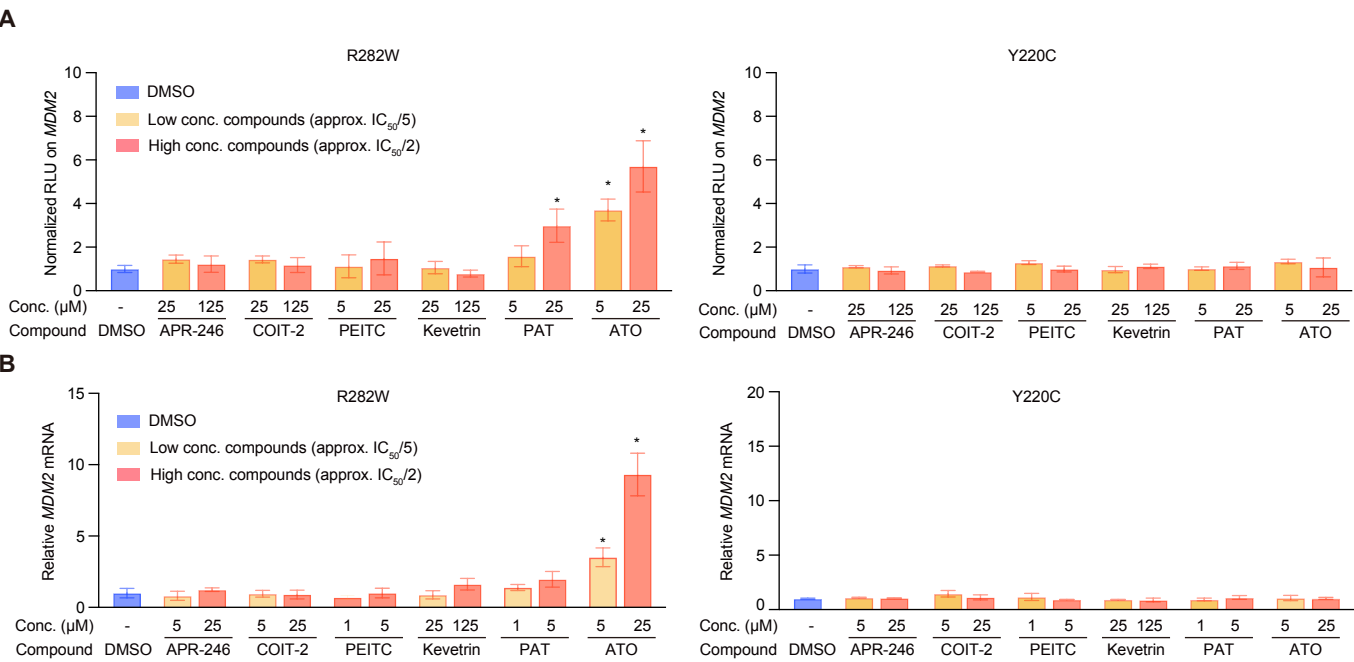

Supplement: Supplementary file 3 — supplemental figure 2 [file 41419_2023_6281_MOESM3_ESM.pdf]

# Supple 3

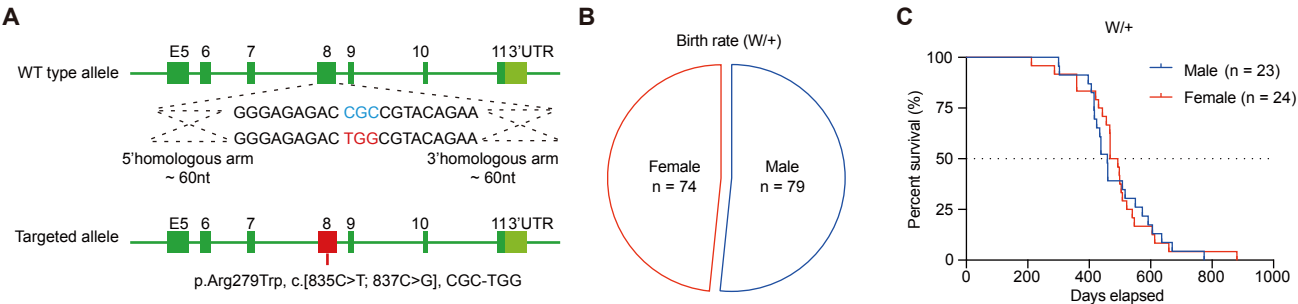

Supplement: Supplementary file 4 — supplemental figure 3 [file 41419_2023_6281_MOESM4_ESM.pdf]

# Supple 4

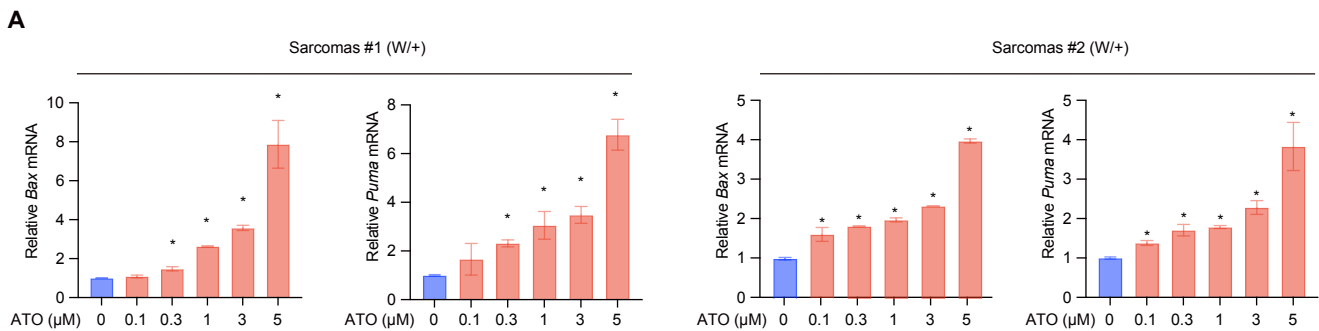

Supplement: Supplementary file 5 — supplemental figure 4 [file 41419_2023_6281_MOESM5_ESM.pdf]

# Supple 5

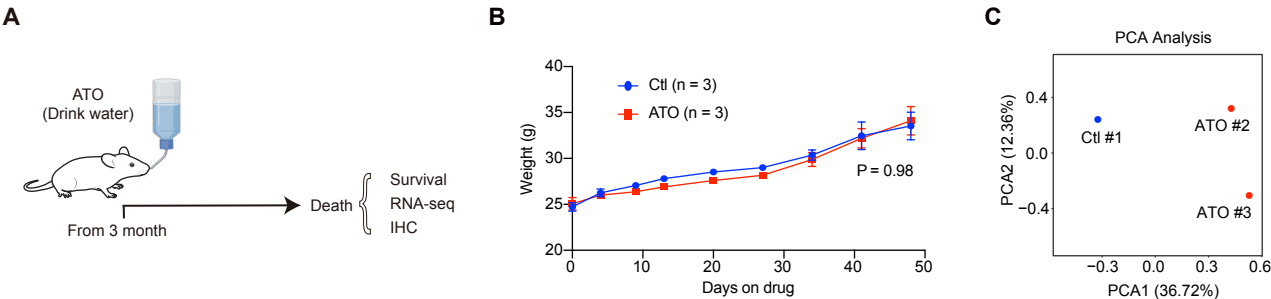

Supplement: Supplementary file 6 — supplemental figure 5 [file 41419_2023_6281_MOESM6_ESM.pdf]

Figure 2C (left)

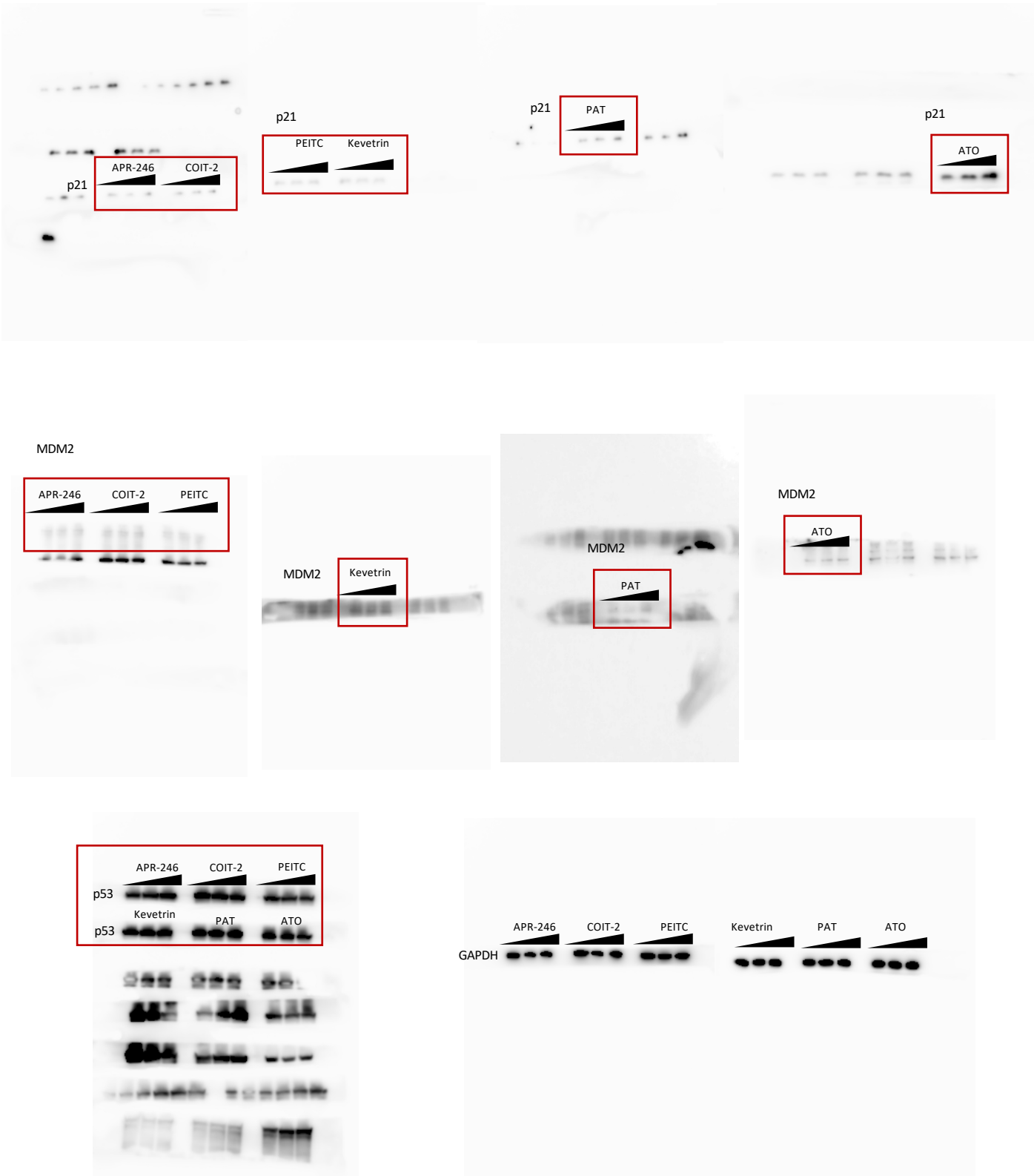

Figure 2C (right)

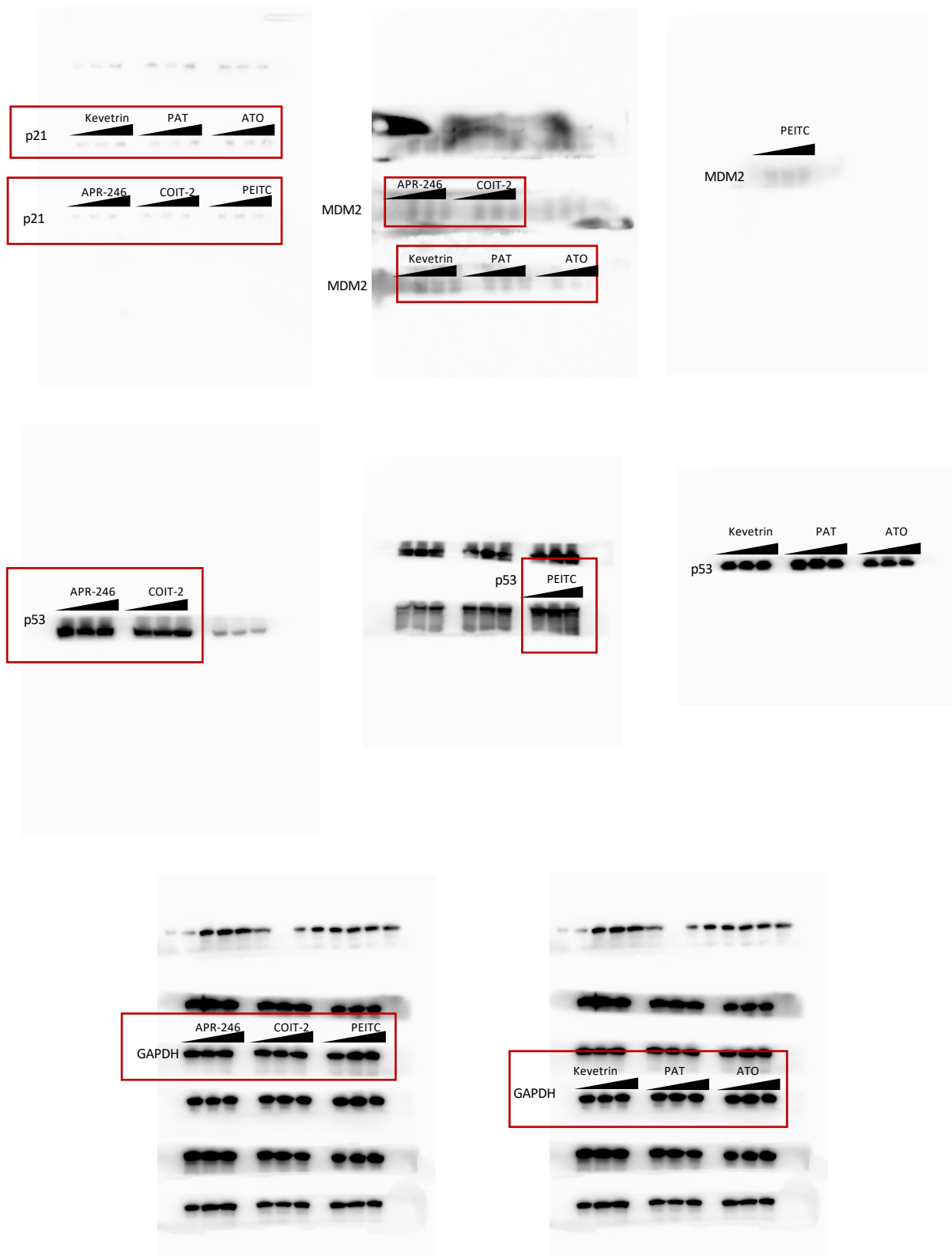

Figure 4B

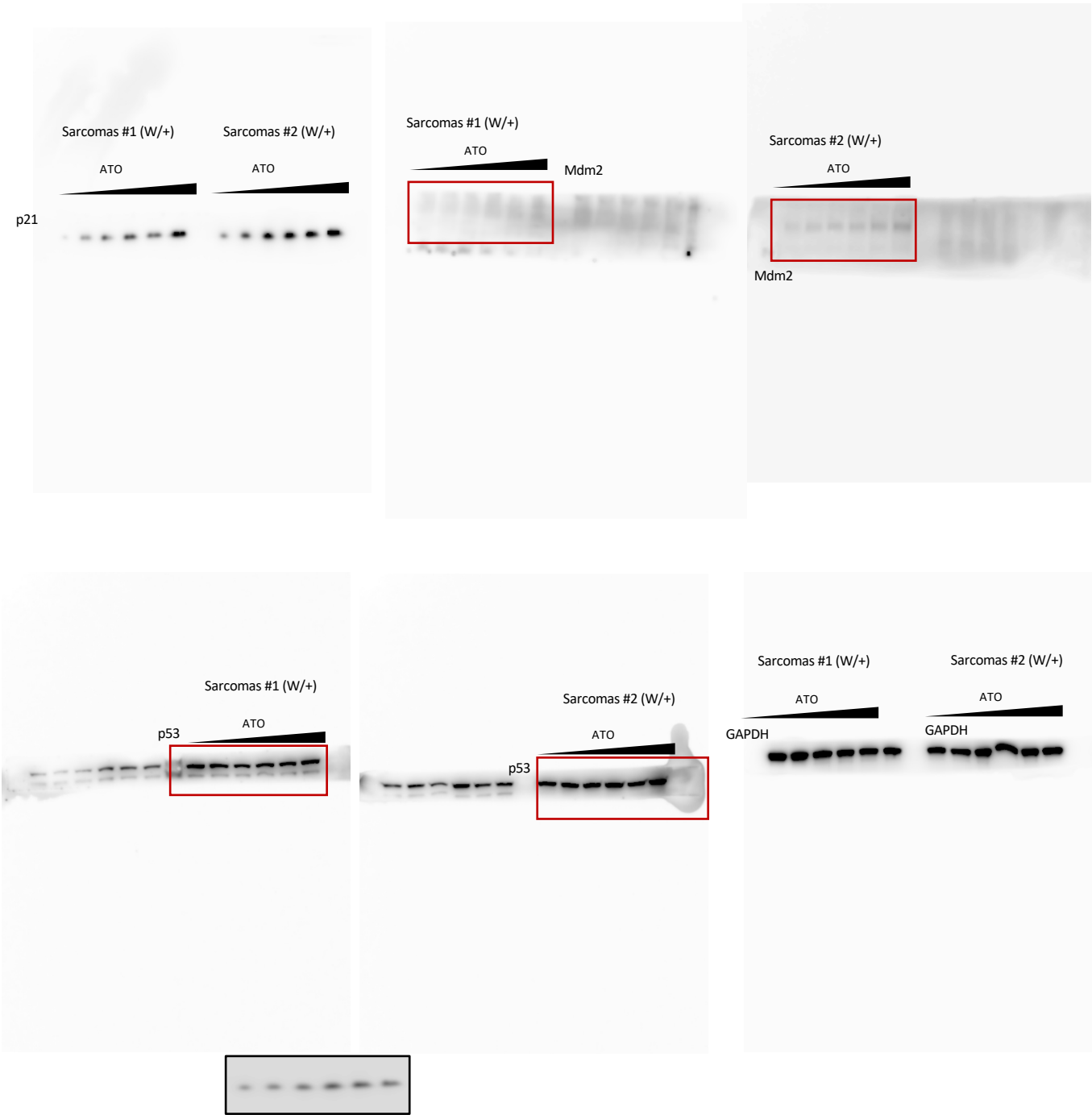

Figure 2C (left)

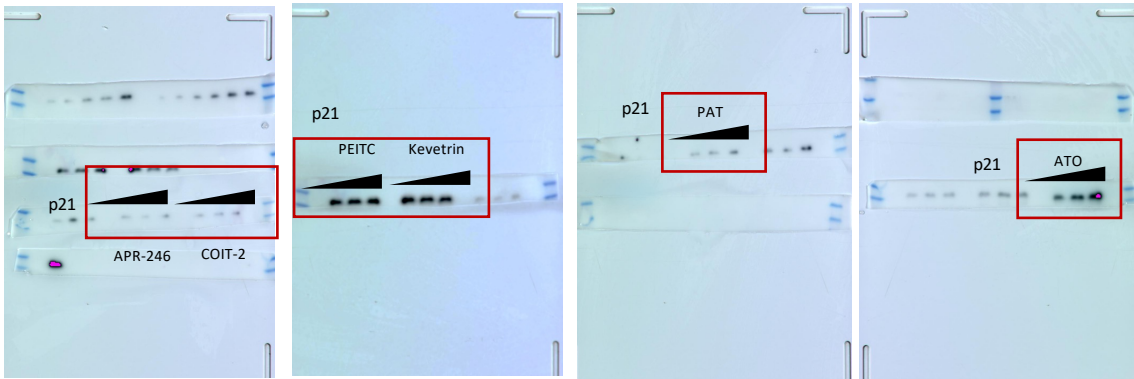

MDM2

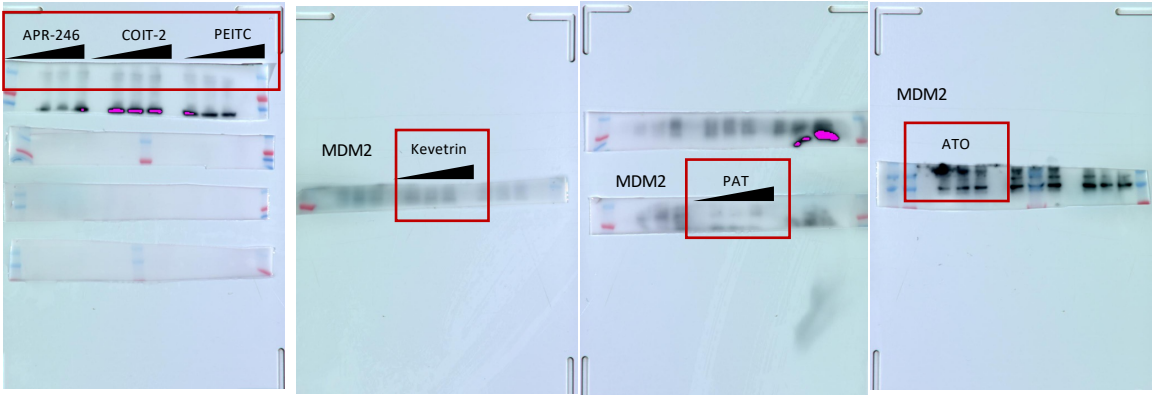

p53

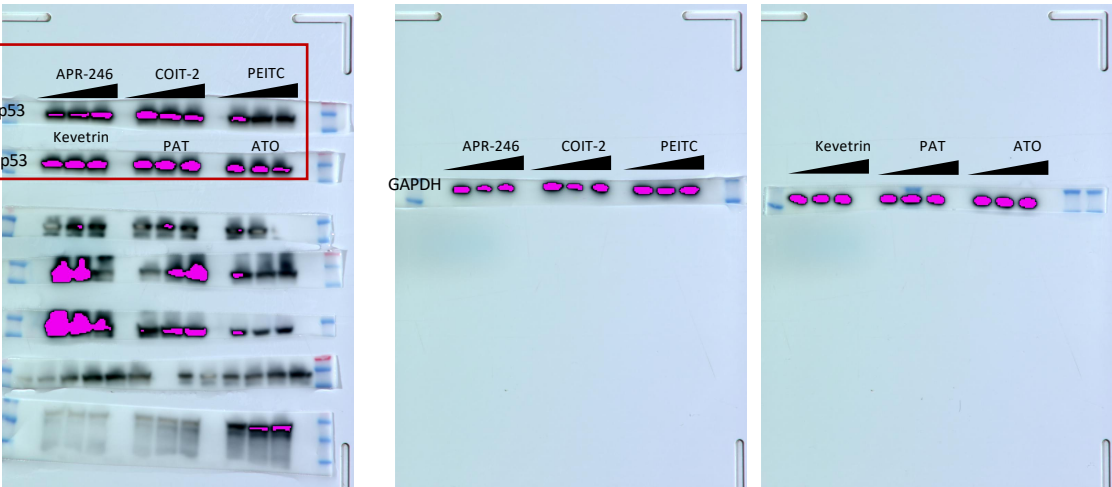

Figure 2C (right)

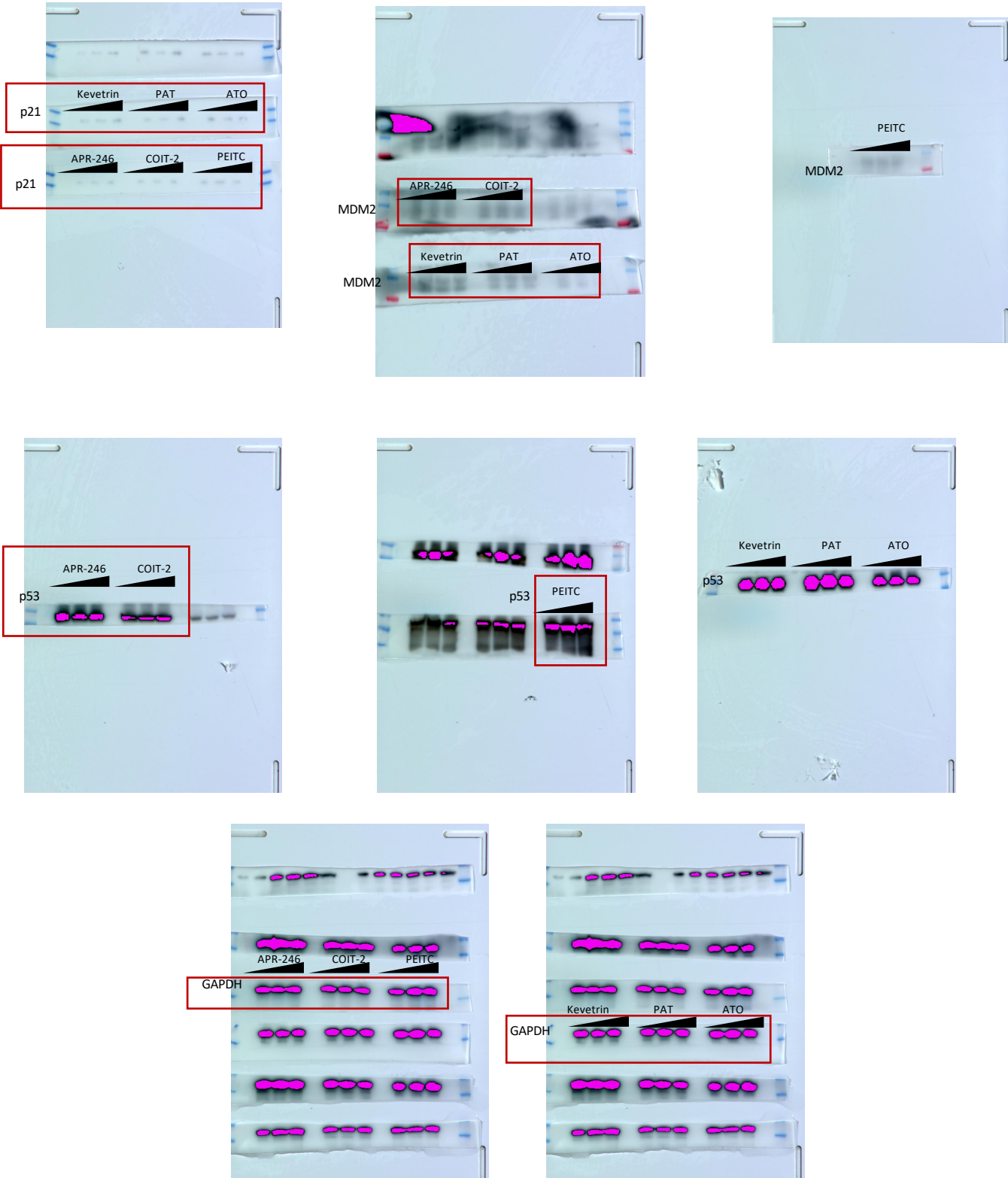

Figure 4B

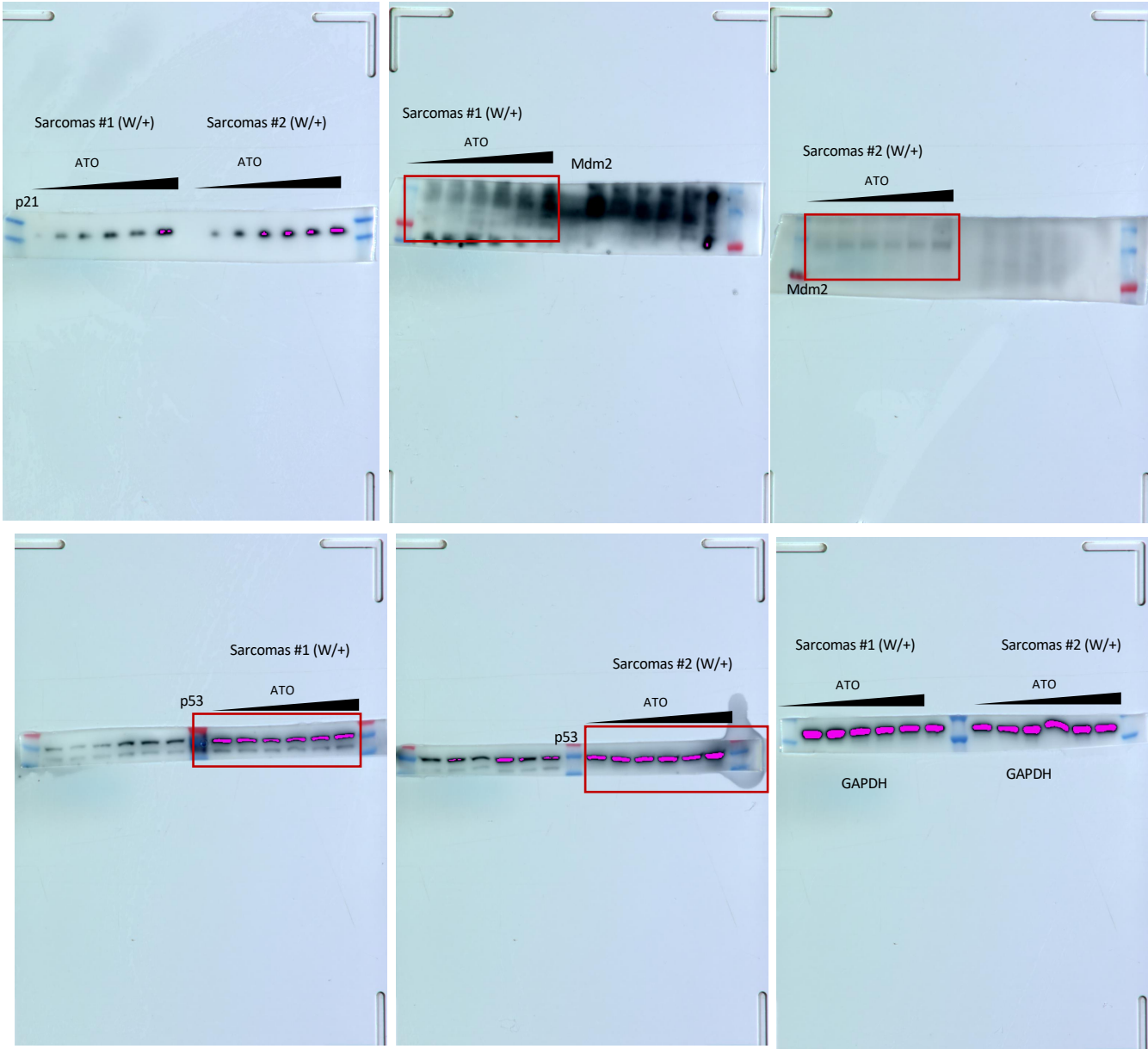

Supplement: Supplementary file 7 — Original Data File [file 41419_2023_6281_MOESM7_ESM.pdf]
